# Total mesorectal excision with or without preoperative chemoradiotherapy for resectable mid/low rectal cancer: a long-term analysis of a prospective, single-center, randomized trial

**DOI:** 10.1186/s40880-018-0342-8

**Published:** 2018-12-20

**Authors:** Fulong Wang, Wenhua Fan, Jianhong Peng, Zhenhai Lu, Zhizhong Pan, Liren Li, Yuanhong Gao, Hui Li, Gong Chen, Xiaojun Wu, Peirong Ding, Zhifan Zeng, Desen Wan

**Affiliations:** 1 Sun Yat-sen University Cancer Center, State Key Laboratory of Oncology in South China; Collaborative Innovation Center for Cancer Medicine, Guangzhou, 510060 Guangdong P. R. China; 2 Department of Colorectal Surgery, Sun Yat-sen University Cancer Center, 651 Dongfeng Road East, Guangzhou, 510060 Guangdong P. R. China; 3 Department of Radiation Oncology, Sun Yat-sen University Cancer Center, Guangzhou, 510060 Guangdong P. R. China; 4 Department of Gynecological Oncology, Sun Yat-sen Memorial Hospital, Sun Yat-sen University, Guangzhou, 510060 Guangdong P. R. China

**Keywords:** Rectal cancer, Total mesorectal excision, Chemoradiotherapy, Long-term outcomes, Phase II randomized trial

## Abstract

**Background:**

The preliminary results of our phase II randomized trial reported comparable functional sphincter preservation rates and short-term survival outcomes between patients undergoing total mesorectal excision (TME) with or without preoperative concurrent chemoradiotherapy (CCRT). We now report the long-term results after a median follow-up of 71 months.

**Methods:**

Between March 23, 2008 and August 2, 2012, 192 patients with T3-T4 or node-positive, resectable, mid/low rectal adenocarcinoma were randomly assigned to receive TME with or without preoperative CCRT. The following endpoints were assessed: cumulative rates of local recurrence and distant metastasis, disease-free survival (DFS), and overall survival (OS).

**Results:**

The data of 184 eligible patients were analyzed: 94 patients in the TME group and 90 patients in the CCRT + TME group. In the whole cohort, the 5-year DFS and OS rates were 84.8% and 85.1%, respectively. The 5-year DFS rates were 85.2% in the CCRT + TME group and 84.3% in the TME group (*P* = 0.969), and the 5-year OS rates were 83.5% in the CCRT + TME group and 86.5% in the TME group (*P* = 0.719). The 5-year cumulative rates of local recurrence were 6.3% and 5.0% (*P* = 0.681), and the 5-year cumulative rates of distant metastasis were 15.0% and 15.7% (*P *= 0.881) in the CCRT + TME and TME groups, respectively. No significant improvements in 5-year DFS and OS were observed with CCRT by subgroup analyses.

**Conclusions:**

Both treatment strategies yielded similar long-term outcomes. A selective policy towards preoperative CCRT is thus recommended for rectal cancer patients if high-quality TME surgery and enhanced chemotherapy can be performed.

*Trial registration* ChiCTR-TRC-08000122. Registered 16 July 2008

## Introduction

Current clinical guidelines suggest that surgical resection still represents the most effective treatment measure for curing patients with mid/low rectal cancer [1, 2]. Total mesorectal excision (TME) has been recognized as the preferred surgical method for resectable rectal cancer, and it reduced locoregional recurrence rates to below 10% and improved cancer-free survival rates to over 70% [3, 4]. To further optimize local treatment, preoperative concurrent chemoradiotherapy (CCRT) consisting of fluoropyrimidine-based chemotherapy and concurrent radiotherapy has been widely performed before TME. Although local recurrence has been significantly reduced to < 7%, 5-year distant metastasis rates are still beyond 20% in mid/low rectal cancer patients after preoperative chemoradiotherapy (CCRT) followed by TME [5–7]. In addition, a certain proportion of patients experience CCRT-related adverse events, including leucopenia, thrombocytopenia, radiation proctitis, anastomotic leakage, and poor wound healing [8–10]. These adverse events can impair the quality of life, lead to great financial burden, and delay subsequent treatment, which might potentially translate into a reduced life expectancy in patients [11–13]. Given these suboptimal treatment outcomes, we considered the survival benefit of preoperative CCRT as questionable for patients with resectable mid/low rectal cancer.

To confirm the prognostic effect of CCRT, we completed a prospective, randomized phase II trial comparing TME with and without preoperative CCRT, both followed by postoperative adjuvant chemotherapy. The short-term results were reported in 2015, and the two groups had similar functional sphincter preservation rates and 3-year survival outcomes [14]. In the absence of a short-term survival benefit, prolongation of follow-up duration was necessary to observe late postoperative recurrences and survival events to obtain a more definite result. Here, we reported the long-term outcomes including local recurrence, distant metastasis, disease-free survival (DFS), and overall survival (OS) from the current trial after a median follow-up of 71 months.

## Patients and methods

### Patient eligibility, randomization, and treatment

The present study was designed as a prospective, randomized phase II trial (Clinical Trial Number ChiCTR-TRC-08000122) approved by the Institutional Research Ethics Committee of Sun Yat-sen University Cancer Center (Approval Number: YP2008005). Informed consent was obtained from the patients before initial treatment. The design of this trial has been previously reported [14]. The eligibility criteria were as follows: (1) histologically confirmed rectal adenocarcinoma; (2) inferior tumor margin within 10 cm from the anal verge before CRT; (3) presence of clinical T3-T4 or node-positive resectable tumor; (4) no malignant disease in the anal canal; and (5) no initial evidence of distant metastasis. All patients were required to undergo colonofiberscopy, endorectal ultrasonography (ERUS), computed tomography (CT) scanning of the chest, abdomen and pelvis, and/or abdominopelvic magnetic resonance imaging (MRI) before CCRT and surgery to determine the resectibility of rectal tumor. Patients were randomly allocated into the TME and CCRT + TME groups in a ratio of 1:1 using a computer generated scheme, and their identities were concealed in consecutively numbered, opaque, sealed envelopes. Patients in the TME group underwent TME followed by 6 cycles of standard XELOX regimen (oxaliplatin at 130 mg/m^2^ on Day 1 and capecitabine at 1000 mg/m^2^ twice daily on Days 1–14 with an interval of 7 days) for pathologic stage II–III disease. Patients in the CCRT + TME group underwent preoperative CCRT (oxaliplatin at 100 mg/m^2^ on Day 1 and capecitabine at 1000 mg/m^2^ twice daily on Days 1–14 with an interval of 7 days with a concurrent total dose of 50 Gy radiotherapy) followed by TME and subsequently 4 cycles of postoperative standard XELOX adjuvant chemotherapy.

### Follow-up

The protocol-specified follow-up was conducted every 3 months for the first 2 years after surgery and every 6 months for the following 3 years. Evaluations consisted of physical examination, complete blood count and blood chemistry analyses, carcinoembryonic antigen (CEA) and cancer antigen 19-9 (CA19-9) measurement, chest radiography, and abdominal ultrasound at each visit. Chest/abdomen/pelvis CT and/or abdominopelvic MRI were performed every 6 months, and colonofiberscopy was performed annually. The final follow-up visit occurred in June 2017.

### Endpoint definition

The primary endpoint was DFS. Secondary endpoints included OS and cumulative rates of local recurrence and distant metastasis. Recurrence within the pelvis was defined as local recurrence, and recurrence outside the pelvis was considered as distant metastasis. Both local recurrence and distant metastasis were considered as postoperative recurrence. OS was defined as the interval from tumor resection to the date of death from any cause or the date of last follow-up, whereas DFS was defined as the interval from tumor resection to the date of disease recurrence, death from disease, or last follow-up. Patients without local recurrence, distant metastasis, or death at the last follow-up date were subjected to random censoring.

### Statistical analysis

All analyses were performed using SPSS statistics software, version 21.0 (IBM Corp., Armonk, NY, USA) and GraphPad Prism version 6.01 (GraphPad Software, Inc., La Jolla, CA, USA). Clinicopathologic parameters were compared between the two treatment groups by using the Chi square test or Fisher’s exact test as appropriate. Survival outcomes were estimated with the Kaplan–Meier method, and differences between groups were assessed with the log-rank test. Variables that were statistically significant with a *P *< 0.05 in univariate Cox models for DFS and OS were further assessed with multivariate Cox models. Hazard ratios (HRs) and 95% confidence intervals (CIs) were calculated. All statistical tests used in this study were two-sided, and a *P* value < 0.05 was considered significant.

## Results

### Patients, tumor characteristics, and treatment compliance

**Fig. 1:**
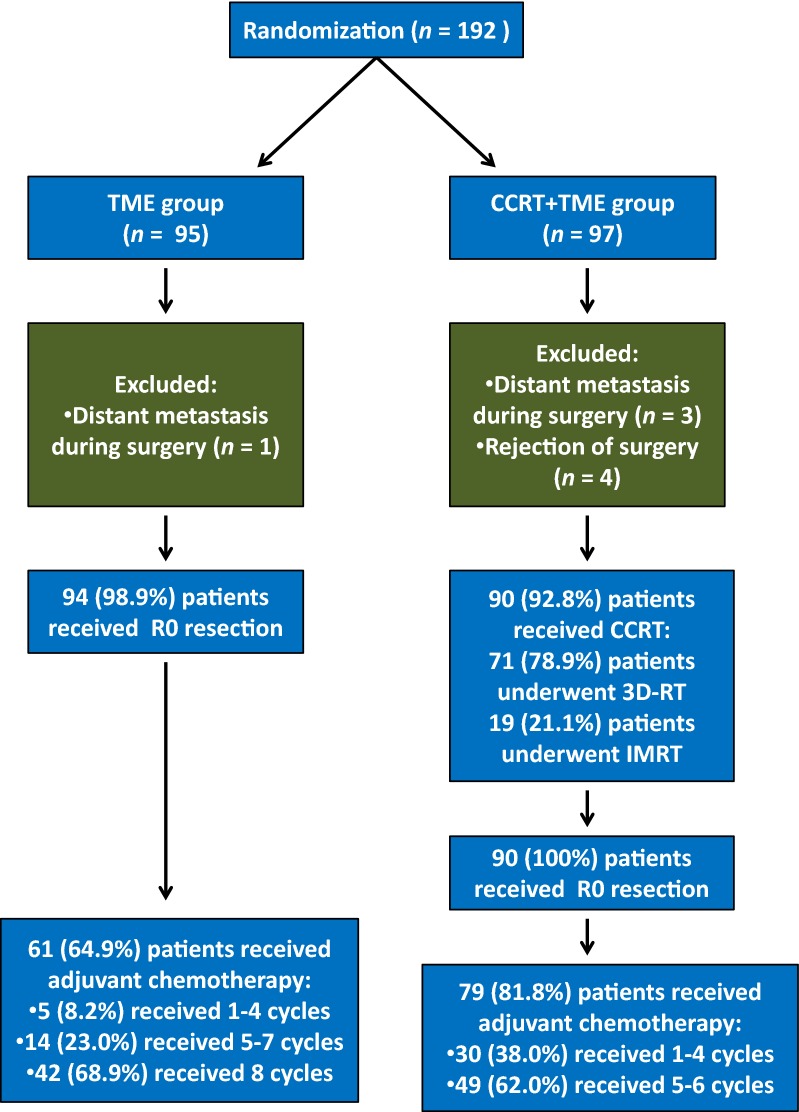
Trial profile of patient eligibility, randomization, and treatment. TME, total mesorectal excision; CCRT, concurrent chemoradiotherapy; 3D-RT, 3-dimentional radiotherapy; IMRT, intensity-modulated radiation therapy

Figure 1 shows the trial profile. Between March 23, 2008 and August 2, 2012, 192 patients were enrolled from Sun Yat-sen University Cancer Center, with 95 patients randomized into the TME group and 97 to the CCRT + TME group. Eight patients were considered ineligible after randomization, and the reasons for ineligibility are summarized in Fig. 1. The number of eligible patients was 94 (98.9%) of 95 in the TME group and 90 (92.8%) of 97 in the CCRT + TME group. Chemotherapy compliance and CCRT safety have been previously reported [14]. All patients underwent R0 resection of rectal tumor. The median ages of the TME group and the CCRT + TME group were 58 years (range 29–70 years) and 56 years (range 28–70 years), respectively. The median numbers of perioperative chemotherapy cycles were 6 (range 0–8) for the TME group and 6 (range 2–8) for the CCRT + TME group. The demographic and treatment characteristics of the TME and CCRT + TME groups were comparable except for pathologic T stage (pT) and adjuvant chemotherapy (both *P* < 0.001) (Table 1).

**Table 1:** Clinicopathologic and treatment characteristics of 184 patients with mid/low rectal cancer

Variable	TME group [cases (%)]	CCRT + TME group [cases (%)]	*P* value
Total	94	90	
Age (years)			
≤ 60	62 (66.0)	64 (71.1)	0.452
> 60	32 (34.0)	26 (28.9)	
Sex			0.273
Male	51 (54.3)	56 (62.2)	
Female	43 (45.7)	34 (37.8)	
DAV (cm)			0.290
≤ 5	47 (50.0)	52 (57.8)	
> 5	47 (50.0)	38 (42.2)	
cTNM stage			0.097
II	48 (51.1)	33 (36.7)	
III	46 (48.9)	57 (63.3)	
Type of resection			0.849
LAR	67 (71.3)	63 (70.0)	
APR	27 (28.7)	27 (30.0)	
pT stage			< 0.001
pT0–2	22 (23.4)	56 (62.2)	
pT3–4	72 (76.6)	34 (37.8)	
pN stage			0.005
pN0	55 (58.5)	70 (77.8)	
pN1–2	39 (41.5)	20 (22.2)	
pTNM stage			< 0.001
pT0N0M0	0	32 (35.6)	
pT1–2N0M0	16 (17.0)	17 (18.9)	
pT3–4N0M0	39 (41.5)	21 (23.3)	
pT1–4N1–2M0	39 (41.5)	20 (22.2)	
Adjuvant chemotherapy			< 0.001
Yes	61 (64.9)	79 (87.8)	
No	33 (35.1)	11 (12.2)	
Cycles of perioperative chemotherapy			
1–7	52 (55.3)	56 (62.2)	0.342
8	42 (44.7)	34 (37.8)	

TME, total mesorectal excision; CCRT, concurrent chemoradiotherapy; DAV, distance of the inferior tumor margin from the anal verge; cTNM stage, clinical tumor-node-metastasis classification; LAR, low anterior resection; APR, abdominoperineal resection; pTNM stage, pathologic tumor-node-metastasis classification; pT stage, pathologic tumor stage; pN stage, pathologic node stage

### Follow-up and postoperative events

**Fig. 2:**
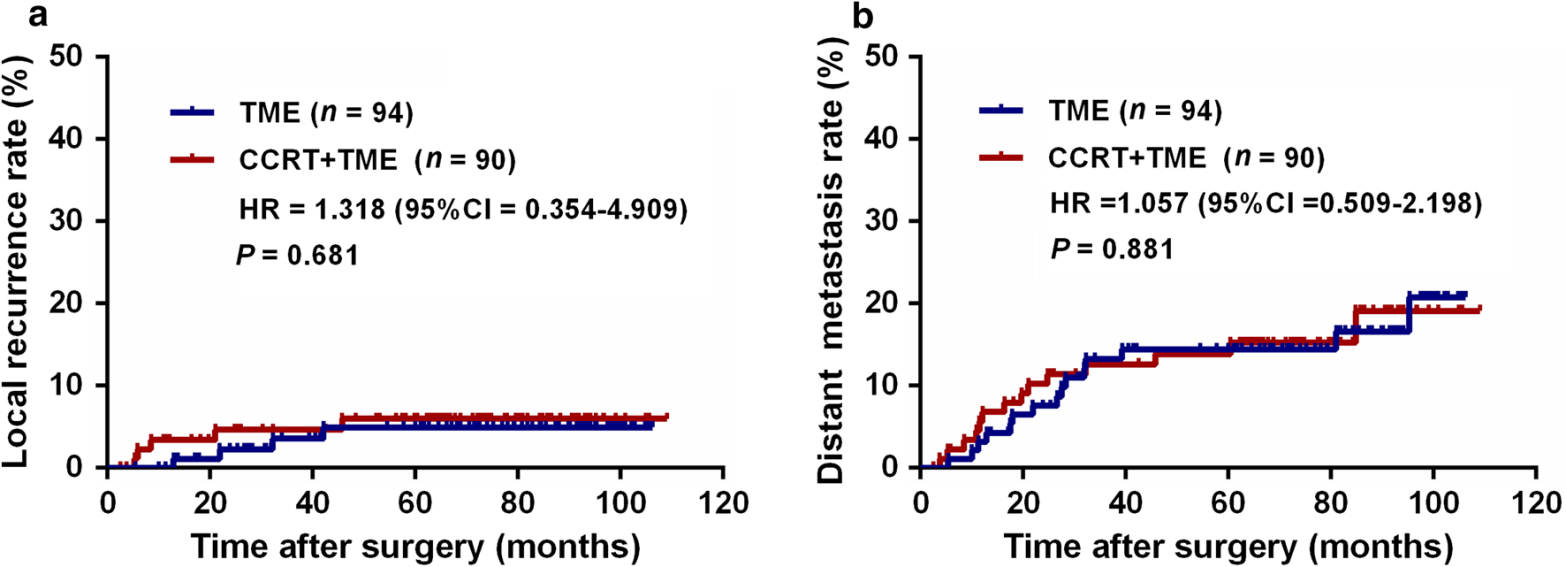
Cumulative rates of local recurrence (**a**) and distant metastasis (**b**) in the TME and CCRT + TME groups. TME, total mesorectal excision; CCRT, concurrent chemoradiotherapy; HR, hazard ratio; CI, confidence interval

At the time of analysis, the median follow-up duration was 71 months (range 4–109 months) for all patients, with 76 months (range 10–106 months) for the TME group and 66 months (range 4–109 months) for the CCRT + TME group. The 5-year survival data were obtained for 83 (88.3%) patients in the TME group and for 80 (88.9%) patients in the CCRT + TME group. A total of 37 (20.1%) patients died during follow-up; among them, 21 were in the TME group, and 16 were in the CCRT + TME group, including 35 who died due to rectal cancer progression, 1 due to nasopharyngeal cancer (NPC), and 1 due to other cause. After tumor resection, local recurrence occurred in 9 (4.9%) patients, whereas 29 (15.8%) developed distant metastasis in the whole cohort. Table 2 shows that the postoperative recurrence periods and patterns were comparable across the two treatment groups. The 5-year cumulative local recurrence rates were 6.3% and 5.0% for the CCRT + TME and TME groups, respectively (HR = 1.318, 95% CI = 0.354–4.909, *P *= 0.681, Fig. 2a). The 5-year cumulative distant metastasis rates were 15.0% and 15.7% for the CCRT + TME and the TME group, respectively (HR = 1.057, 95% CI = 0.509–2.198, *P *= 0.881, Fig. 2b).

**Table 2:** Recurrence after radical resection in the TME and CCRT + TME groups

Variable	TME group [cases (%)]	CCRT + TME group [cases (%)]	*P* value
Postoperative recurrence			0.971
Yes	19 (20.2)	18 (20.0)	
No	75 (79.8)	72 (80.0)	
Recurrence period			0.858
Early recurrence (≤ 24 months)	10 (52.6)	10 (55.6)	
Late recurrence (> 24 months)	9 (47.4)	8 (44.4)	
Recurrence pattern^a^			
Local recurrence	4 (4.3)	5 (5.6)	0.743
Liver metastasis	2 (2.1)	4 (4.4)	0.437
Lung metastasis	9 (9.6)	7 (7.8)	0.665
Other site metastasis	4 (4.3)	6 (6.7)	0.530

TME, total mesorectal excision; CCRT, concurrent chemoradiotherapy

^a^There were 3 patients developed multiple metastasis

### Overall and disease-free survival

**Fig. 3:**
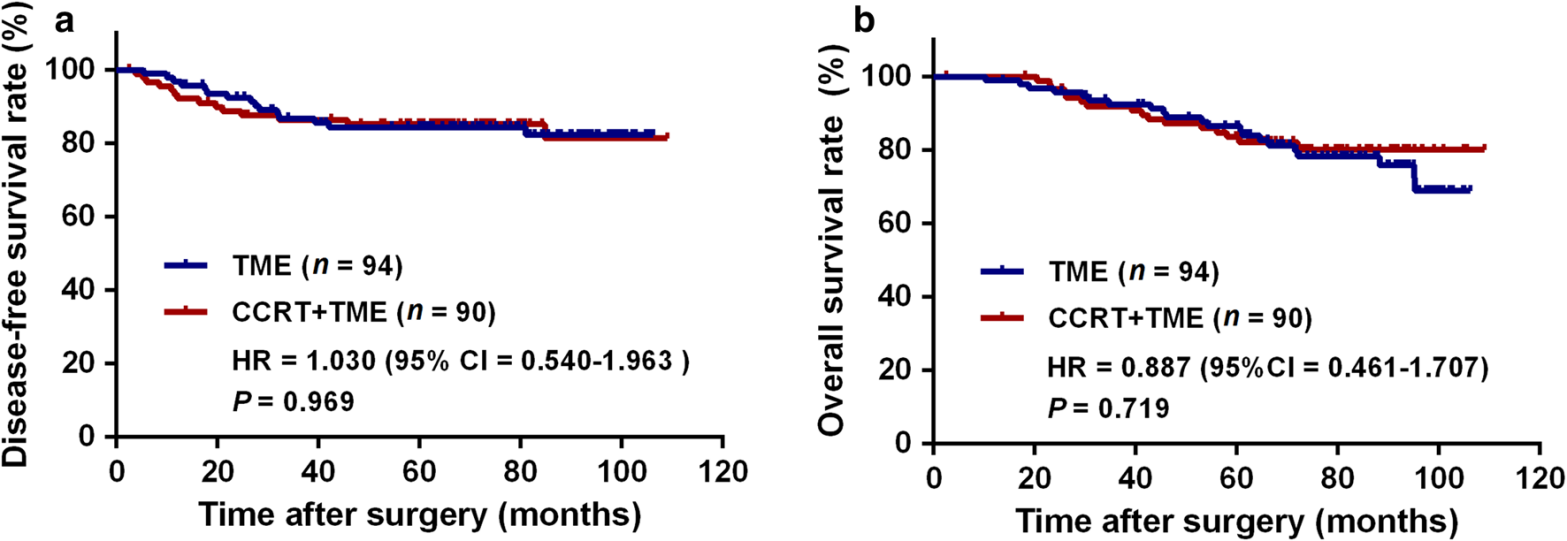
Disease-free survival (**a**) and overall survival curves (**b**) of the TME and CCRT + TME groups. TME, total mesorectal excision; CCRT, concurrent chemoradiotherapy; HR, hazard ratio; CI, confidence interval

In the whole cohort, the 5-year DFS and OS rates were 84.8% and 85.1%, respectively. There were no significant differences in the 5-year DFS and OS rates between the CCRT + TME and TME groups (5-year DFS rate: 85.2% vs. 84.3%, *P* = 0.969, Fig. 3a; 5-year OS rate: 83.5% vs. 86.5%, *P *= 0.719, Fig. 3b). Univariate analysis revealed that pT3–4 (HR = 2.940, 95% CI = 1.344–6.434, *P *= 0.007) and pathologic N stage (pN) 1–2 (HR = 2.903, 95% CI = 1.519–5.546, *P* = 0.001) were significant negative predictors of 5-year DFS rate. In the multivariate Cox proportional hazards model, pN1–2 (HR = 2.281, 95% CI = 1.157–4.495, *P *= 0.017) was identified as an independent predictor of 5-year DFS rate. For 5-year OS rate, pT3–4 (HR = 2.365, 95% CI = 1.115–5.018, *P* = 0.025) was the only significant negative predictor (Table 3).

**Table 3:** Univariate and multivariate analyses of prognostic factors for disease-free survival and overall survival in 184 patients with mid/low rectal cancer

Variable	5-year DFS rate				5-year OS rate	
	Univariate		Multivariate		Univariate	
	HR (95% CI)	*P* value	HR (95% CI)	*P* value	HR (95% CI)	*P* value
Age (> 60 vs. ≤ 60 years)	0.545 (0.249–1.194)	0.129			0.862 (0.425–1.748)	0.681
Sex (male vs. female)	0.878 (0.460–1.677)	0.694			0.758 (0.397–1.444)	0.399
DAV (≤ 5 cm vs. > 5 cm)	0.698 (0.361–1.315)	0.259			0.834 (0.488–1.783)	0.834
pT stage (3–4 vs. 0–2)	2.940 (1.344–6.434)	*0.007*	2.242 (0.987–5.093)	0.054	2.365 (1.115–5.018)	*0.025*
pN stage (1–2 vs. 0)	2.903 (1.519–5.546)	*0.001*	2.281 (1.157–4.495)	*0.017*	1.801 (0.939–3.453)	0.076
Type of resection (LAR vs. APR)	0.983 (0.485–1.989)	0.961			0.693 (0.356–1.348)	0.280
Perioperative chemotherapy cycles (≤ 6 vs. > 6)	2.124 (0.971–4.646)	0.059			1.381 (0.682–2.798)	0.370
Treatment (CCRT + TME vs. TME)	1.030 (0.540–1.963)	0.929			0.887 (0.461–1.707)	0.720

DFS, disease-free survival; OS, overall survival; HR, hazard ratio; CI, confidence interval; DAV, distance of the inferior tumor margin from the anal verge; pT stage, pathologic tumor stage; pN stage, pathologic node stage; LAR, low anterior resection; APR, abdominoperineal resection; TME, total mesorectal excision; CCRT, concurrent chemoradiotherapy

Italic values indicate significance of *p* vaule (*p* < 0.05)

**Fig. 4:**
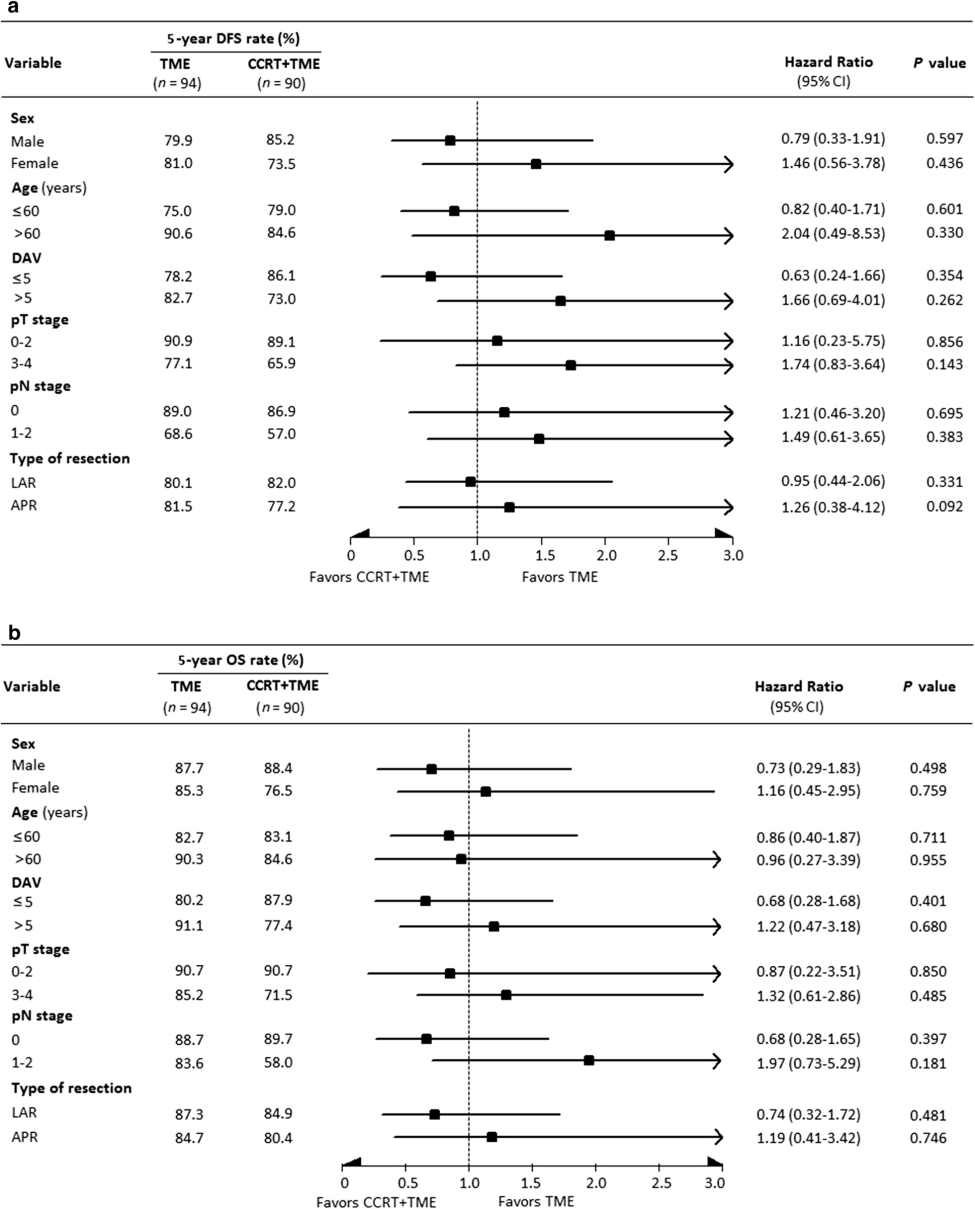
Forest plot of disease-free survival (**a**) and overall survival (**b**) of the TME and CCRT + TME groups in stratification analyses. TME, total mesorectal excision; CCRT, concurrent chemoradiotherapy; DFS, disease-free survival; OS, overall survival; CI, confidence interval; LAR, low anterior resection; APR, abdominoperineal resection

Figure 4 shows a forest plot with HR for 5-year DFS and OS rates in the CCRT + TME group compared with the TME group stratified by sex, age, distance of the inferior tumor margin from the anal verge, pT and pN stages, and type of tumor resection. The results showed that the 5-year DFS and OS rates were comparable between the two groups in all stratification analyses.

## Discussion

The current study reveals that after a median follow-up of 69 months, preoperative CCRT followed by TME yields similar 5-year survival outcomes, including DFS, OS, cumulative local recurrence, and distant metastasis, to TME alone. These findings suggest that the widespread application of preoperative CCRT might not achieve expected survival benefits if high-quality TME surgery can be performed.

It is well known that compared with TME alone, preoperative radiotherapy improves local disease control for locally advanced rectal cancer, with a relative risk reduction of greater than 50% for postoperative local recurrence [15, 16]. However, our results showed that the 5-year cumulative rate of local recurrence was only slightly higher in the CCRT + TME group than in the TME group (6.3% vs. 5.0%, *P *= 0.681). Similarly, Williamson et al. [6] reported that preoperative CRT followed by surgery resulted in a higher 5-year local recurrence rate than did surgery alone for patients with stage II/III mid/low rectal cancer (6.5% vs. 0, *P* = 0.040). Because high-quality surgery resulted in a local recurrence rate of less than 5%, the significance of benefits from preoperative CCRT were difficult to determine for this selective cohort. Moreover, a trial involving TME conducted by the Dutch Colorectal Cancer Group (DCCG) has noted that the superiority of preoperative radiotherapy on local recurrence reduction was decreased from 70.7% at 2 years to 48.6% at 5 years during follow-up [15, 17]. Even after 12 years of follow-up, the rate of local recurrence was similar to that at 5 years after surgery, with 11% in the surgery-alone group and 9% in the radiotherapy + surgery group [18], indicating that the effect of radiotherapy on local recurrence control may be attenuated with time.

Consistent with our findings, previous clinical trials have demonstrated that distant metastasis rate was 3–6 times higher than the local recurrence rate in locally advanced rectal cancer, and metastasis remains the predominant reason for treatment failure [19–21], probably because of insufficient systemic control of micrometastasis by chemotherapy. To enhance systemic control, we added oxaliplatin to the capecitabine-based (XELOX) regimen for the CCRT + TME group, which has been shown to result in a significant increase in tumor response and to be well tolerated in Chinese locally advanced rectal cancer patients [22–24]. Although the CCRT + TME group achieved a 35.6% pathologic complete response (pCR) rate in our previous analysis [14], this treatment strategy failed to further reduce the distant metastasis rate relative to that in the TME group (3-year: 10.0% vs. 12.8%, *P *= 0.834; 5-year: 15.0% vs. 15.7%, *P *= 0.881). To the best of our knowledge, the value of adding oxaliplatin to the preoperative CCRT regimen for controlling distant metastasis remains unconfirmed [25–27]. Therefore, use of oxaliplatin may be debatable for these patients.

Thus far, accumulating evidence after long-term follow-up has revealed that preoperative CCRT had no significant effect on prolonging OS or DFS in patients with resectable rectal cancer [6, 28, 29]. In line with the 3-year survival outcomes of our patients [14], no significant differences were observed for the 5-year DFS and OS rates between the CCRT + TME and TME groups in the present study. It should be emphasized that perioperative chemotherapy with the XELOX regimen was recommended for all patients in the present study. Adjuvant chemotherapy with the XELOX regimen has been demonstrated to improve long-term survival in patients with resectable stage III colon cancer, with a potential effect on eliminating micrometastatic disease [30–32]. In the present study, both groups of patients received a median of 6 cycles of perioperative chemotherapy, and the completion rate of full course of therapy was comparable between the two groups (44.7% vs. 37.8%, *P *= 0.342). Based on these results, the efficacy of perioperative chemotherapy might outweigh the effect of CCRT on survival outcomes. Therefore, 6–8 cycles of perioperative chemotherapy with the XELOX regimen should be recommended for rectal cancer patients, irrespective of whether they undergo TME alone or in combination with CCRT, to minimize the risk of tumor recurrence.

Tumor pathologic stage has been widely confirmed as a prominent factor affecting survival outcomes in rectal cancer patients [33, 34]. In the present study, pN1–2 and pT3–4 were identified as the most important risk factors for 5-year DFS and OS rates, respectively, indicating that when high-quality surgery could be performed, the prognosis was not determined by preoperative treatment strategy but rather by tumor parameters. We also attempted to identify subgroups of patients with specific tumor features who would benefit from preoperative CCRT. Unfortunately, none of the subgroups of patients displayed significant improvements in 5-year survival outcomes after treatment with preoperative CCRT in the present study. The DCCG TME trial [17, 18] and a Swedish rectal cancer trial [35] have shown that the effects of preoperative radiotherapy were most convincing for patients with TNM stage III, mid/low rectal cancer without circumferential resection margin involvement. Because the high quality of TME and the systemic treatment with chemotherapy are likely to result in noticeable effects on survival, our trial was not able to capture the small but potential meaningful differences between the TME and CCRT + TME groups.

Some limitations of this study should be acknowledged. Because new diagnostic techniques, including MRI and ERUS, were not available at our center between 2008 and 2009, tumor staging was mostly based on CT scan examination, which explains the 62.8% and 41.5% staging accuracy for pT and pN stage, respectively [14]. This limitation might have caused a certain proportion of patients with stage I disease to be overtreated with preoperative CCRT, which might have led to the underestimation of the effect of preoperative CCRT. In addition, the recently identified high-risk parameters for rectal cancer such as threatened mesorectal fascia and extramural venous invasion were not evaluated by high-resolution MRI in the present study. These parameters might help to further optimize preoperative treatment strategies [36, 37]. Moreover, data on the molecular characteristics of tumors were not available for the patients in the present study. It has been shown that the expression of cyclooxygenase-2, vascular endothelial growth factor, and Raf kinase inhibitor protein and the mutational status of KRAS represent valuable molecular markers that can be used for predicting treatment outcomes in rectal cancer patients [38, 39]. Therefore, future studies should include the analysis of tumor molecular biomarkers.

## Conclusions

Despite the increase in follow-up durations, there is still no conclusive effect of CCRT on survival outcomes. Our long-term data supported that a selective policy towards preoperative CCRT should be practiced for rectal cancer patients if high-quality TME surgery and aggressive chemotherapy were performed.
